# Multisite Dopamine Sensing With Femtomolar Resolution Using a CMOS Enabled Aptasensor Chip

**DOI:** 10.3389/fnins.2022.875656

**Published:** 2022-06-03

**Authors:** Violetta Sessi, Bergoi Ibarlucea, Florent Seichepine, Stephanie Klinghammer, Imad Ibrahim, André Heinzig, Nadine Szabo, Thomas Mikolajick, Andreas Hierlemann, Urs Frey, Walter M. Weber, Larysa Baraban, Gianaurelio Cuniberti

**Affiliations:** ^1^Institute of Semiconductor and Microsystems, TU Dresden, Dresden, Germany; ^2^Center for Advancing Electronics Dresden, TU Dresden, Dresden, Germany; ^3^Max Bergman Center of Biomaterials Dresden and Institute for Materials Science, TU Dresden, Dresden, Germany; ^4^RIKEN Quantitative Biological Center, Kobe, Japan; ^5^Imperial College London, London, United Kingdom; ^6^NaMLab gGmbH, Dresden, Germany; ^7^Department of Biosystems Science and Engineering, Bio Engineering Laboratory, ETH Zürich, Basel, Switzerland; ^8^MaxWell Biosystems AG, Basel, Switzerland; ^9^Institute of Solid State Electronics, TU Wien, Vienna, Austria; ^10^Helmholtz-Zentrum Dresden-Rossendorf e.V., Institute of Radiopharmaceutical Cancer Research, Dresden, Germany

**Keywords:** dopamine detection, silicon nanowire, CMOS (complementary metal oxide semiconductor), aptasensor, multisite array

## Abstract

Many biomarkers including neurotransmitters are found in external body fluids, such as sweat or saliva, but at lower titration levels than they are present in blood. Efficient detection of such biomarkers thus requires, on the one hand, to use techniques offering high sensitivity, and, on the other hand, to use a miniaturized format to carry out diagnostics in a minimally invasive way. Here, we present the hybrid integration of bottom-up silicon-nanowire Schottky-junction FETs (SiNW SJ-FETs) with complementary-metal–oxide–semiconductor (CMOS) readout and amplification electronics to establish a robust biosensing platform with 32 × 32 aptasensor measurement sites at a 100 μm pitch. The applied hetero-junctions yield a selective biomolecular detection down to femtomolar concentrations. Selective and multi-site detection of dopamine is demonstrated at an outstanding sensitivity of ∼1 V/fM. The integrated platform offers great potential for detecting biomarkers at high dilution levels and could be applied, for example, to diagnosing neurodegenerative diseases or monitoring therapy progress based on patient samples, such as tear liquid, saliva, or eccrine sweat.

## Introduction

During Parkinson’s disease development, specific nerve cells (neurons) in the brain gradually break down or die. Many of the symptoms are due to a loss of neurons that produce a chemical messenger called dopamine (DA) (Bernheimer et al., 1973; Aarts et al., 2012). The decrease of DA levels causes abnormal brain activity, leading to impaired movement and other symptoms of Parkinson’s disease. DA therefore has a crucial function in diagnostics and therapy prognosis of Parkinson’s disease. It circulates through the human body in different biofluids but at different concentration levels, generally in the nanomolar range or below (Hou et al., 2010; Diaz-Diestra et al., 2017). In blood or plasma it can be found at picomolar concentrations (Zeng and Jose, 2011; Robertson et al., 2012), while in certain organs like the kidney (Harris and Zhang, 2012) or the brain (Si and Song, 2018) DA reaches nanomolar levels (Bernheimer et al., 1973; Yates et al., 1979; Starr, 1996; Andréa and Coquerel, 2003; Wu et al., 2012; Cordeiro et al., 2017; Dirkx et al., 2017).

Recent clinical demonstrations that neurological-disease markers like DA and other hormones can be found in sweat and saliva, fueled research in the fields of biosensorics and point-of care diagnostics (Yoon et al., 2016; Ventura et al., 2017; Steckl and Ray, 2018; Melanie Rudolph et al., 2019; Klinghammer et al., 2020; Lei et al., 2020; Torrente-Rodríguez et al., 2020). Hormones, such as steroids (cortisol), or catecholamines (DA), are some of the target biomarkers that have great relevance and importance for rapid and sensitive detection by means of miniaturized ultrasensitive platforms. When body fluids are used for biomarker testing, they are commonly diluted to obtain sufficiently large sample volumes, which, in turn, makes it necessary to use highly sensitive techniques that enable measurements at pico- or femtomolar levels. This holds particularly true for body fluids, which may contain lower biomarker levels compared to blood and that are only available in small quantities, like tears or eccrine sweat. Recent developments in the field of chip-based medical screening and diagnostics, therefore, focus on small sensor devices that are reliable and can provide high throughput and immediate readout. Several strategies are being evaluated to push the limits of existing transducers in terms of tradeoff between resolution, noise, speed of response, multiplexed detection as well as fabrication costs (Hierlemann and Gutierrez-Osuna, 2008; Hong et al., 2018). Regarding the detection and sensing of DA, amperometric methods are most commonly employed for *in vitro* studies (Jackowska and Krysinski, 2012). However, potentiometric ion-sensitive field-effect transistors (ISFETs) offer many advantages, since ISFETs do not suffer from increased impedance at small dimensions and enable to benefit from co-integration of multiple sensors on a small footprint. A unique opportunity arises from hybrid silicon-nanowire (SiNW)/CMOS platforms, offering high sensitivity and high spatial resolution superseding those of currently available techniques. The use of nanometer-scale semiconductor materials, such as SiNWs, enables DA detection in the femtomolar range (Lin et al., 2008). SiNWs and related hetero-junctions are particularly promising device candidates as they feature large a surface-to-volume ratio in combination with a high interface gate oxide quality, which allows for sensing low analyte concentrations (Patolsky et al., 2004). SiNW-based devices, functionalized with dedicated receptors, proved capable to selectively detect a range of analytes, (Baraban et al., 2019) such as DNA (Li et al., 2004), proteins (Zheng and Lieber, 2011), cells (Jiang et al., 2012; Fu et al., 2014), or microscopic emulsion droplets (Schütt et al., 2016). The demonstrated sensitivity in many cases has achieved the femtomolar (Puppo et al., 2013, 2014; Shalev et al., 2013; Ibarlucea et al., 2018) or even attomolar levels (Patolsky et al., 2006b; Buitrago et al., 2014; Tzouvadaki et al., 2016), with an ultimate resolution of single-analyte binding events (Patolsky et al., 2004). The published works have shown that detection in hostile environments such as breast cancer tissue (Tuoheti et al., 2020), blood plasma (Nguyen-Le et al., 2022), or saliva (Klinghammer et al., 2020) is possible. The latter aimed at cortisol hormones as target, showing that such platforms hold great promise for monitoring DA in other body fluids.

Most of the SiNW-biosensor works to date have been dedicated to proof-of-principle studies with individual sensor devices (Li et al., 2013), or only included relatively small arrays of discrete transducers [e.g., 1 × 50 in Patolsky et al. (2006a) and 4 × 4 in Qing et al. (2010)]. Biosensing platforms based on top-down CMOS fabrication methods have been reported. While an impressive number of sensor sites and transistor counts have been demonstrated in planar ISFET platforms (Rothberg et al., 2011; Duarte-Guevara et al., 2017; Hu et al., 2017), the corresponding readout or amplification (Duarte-Guevara et al., 2017) circuitry has only been implemented off-chip. This issue was addressed by co-integration of sensors and amplification circuits built from meander-shaped SiNW devices (Huang et al., 2013). Although high analyte sensitivity was confirmed, the large active area (0.625 μm × 2.5 mm), and the low device count per chip, entailed a low spatial resolution. Additionally, the larger sensing area of the devices [of ∼2.5 μm × 2.5 μm 16 and 50 μm × 50 μm (Hu et al., 2017)] reduces the limit of detection and sensitivity of the measurements, compared to the results delivered by the nanoscale transducers (Dorvel et al., 2012).

A promising, alternative approach offering scalability while maintaining high nanoscopic sensitivity includes to combine conventional top-down microfabrication methods with independently fabricated, bottom-up nanostructures through hybrid integration (Livi et al., 2015; Hong et al., 2016; Seichepine et al., 2017; Dudina et al., 2019) a process in which nanostructure fabrication and assembly is decoupled from the microelectronic-chip fabrication. Such an approach gives access to large arrays of highly sensitive nanometer-scale sensing structures, which cannot be fabricated by conventional microelectronic fabrication processes.

**FIGURE 1:**
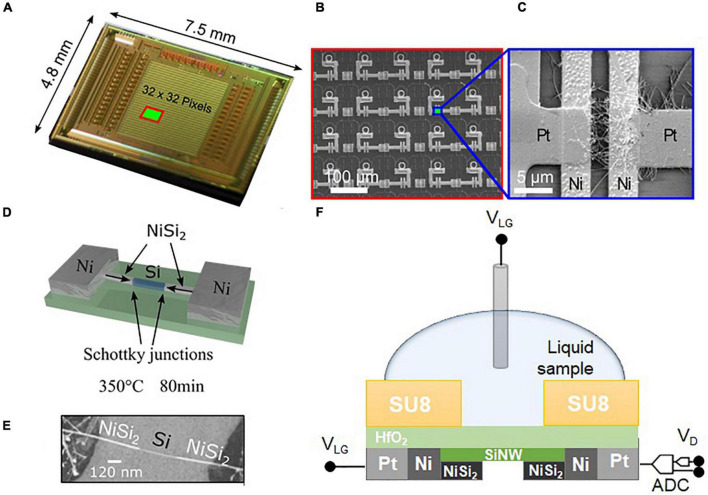
**(A)** Micrograph of the SiNW-CMOS chip. **(B)** SEM image of the SiNW SJ-FET array after connecting the floating lid to the CMOS access points, for a chip area as indicated in panel **(A)**. **(C)** SEM image of one single device showing a bundle of SiNWs captured between FLO and COM and clamped by Ni contacts. **(D)** Schematic view of a device during nickel silicide formation by annealing at 350°C. **(E)** SEM image of the silicide formation along a single SiNW fabricated on an Si/SiO_2_ substrate under the same experimental conditions as the SiNW SJ-FETs integrated in the CMOS chip. The nano-Schottky junctions within the nanowires are formed by the metallic NiSi_2_ segments with atomically abrupt and flat junctions towards the pristine SiNW. **(F)** Cross section of an individual SiNW SJ-FET sensing device with liquid gate.

In this work, the hybrid integration of bottom-up silicon-nanowire Schottky-junction FETs (SiNW SJ-FETs) with CMOS readout and amplification electronics (see Figure 1) is employed to establish a robust high spatio-temporal-resolution biosensing platform with 32 × 32 measurement sites at 100 μm pitch. The combination of CMOS technology, which enables the realization and simultaneous readout of large numbers of devices, with the extraordinary sensing properties of SiNWs provides highly sensitive analyte detection and yields enough sensor data for extended statistical analysis. Moreover, it enables to use a subset of the sensor devices for reference or control measurements and self-validation and to incorporate many different bioreceptors on a very small footprint. Different to the surface-potential-displacement-induced threshold voltage shift in ISFETs, the current injection of our nanojunction sensor units is altered by various orders of magnitude upon binding of analytes. The biosensing capabilities of our devices are demonstrated through specific detection of the neurotransmitter DA at concentrations in the femtomolar range.

## Materials and Methods

### Silicon-Nanowire Growth

Silicon-nanowires were formed by using a Vapor-Liquid-Solid (VLS) growth mechanism in a Chemical Vapor Deposition (CVD) furnace, using synthesized catalytic gold nanoparticles with 20 nm average diameter, pre-deposited on a p-doped Si growth substrate that was covered by native SiO_2_. The gas precursor was mono-silane SiH_4_, and the CVD process temperature and time were 450°C and 40 min, respectively. These parameters resulted in SiNWs with predominant single crystal <1 1 2> axis orientation, wire lengths of approx. 30 μm, and average diameters of 20 nm. After NW growth the Au nanoparticles were removed by immersion in Aqua Regia for 2 h. More details on NW growth are given in Pregl et al. (2013). The grown SiNWs had a crystalline Si core, surrounded by an amorphous shell of native SiO_2_, which formed upon exposure to air.

The NWs were removed from the growth substrate by a short ultra-sonic agitation (5–10 s) in isopropanol. The NW suspensions were then used for dispersing NWs on the CMOS chips as described in the Supplement for further processing or on test substrates for analysis.

### Silicon-Nanowire Sensor Fabrication

The CMOS system was fabricated using an industrial 0.35 μm, double polysilicon process having 4 metal layers (X-FAB, Erfurt, Germany) (Rothe et al., 2014). In addition to the foundry process, a stack of SiO_2_/Si_3_N_4_ layers was added for passivation and protecting the CMOS during operation in the liquid phase, while single access points to the underlying electrode contacts were opened by reactive-ion etching.

In the first step of the nanowire integration, small bundles of pre-synthetized, nominally undoped SiNWs were assembled in pre-defined positions by dielectrophoresis (Figure 1B; Collet et al., 2015). The applied DEP process simultaneously assembled the NWs in 1024 locations. A floating electrode was implemented, according to a fabrication route introduced by Seichepine et al. for CNT devices (Seichepine et al., 2017), with the aim to achieve large-scale fabrication of independent devices in a single step. An electrode geometry with flat ends was chosen to obtain multiple nanowires, connected in parallel per device, in order to increase the active region and the total current while keeping the nanoscopic nature of the nanowires. The process yielded approx. 90% of functional devices in which SiNWs connected the contacts.

In the second step of the integration, nanoscopic source/drain Schottky contacts were fabricated. To form the nano-Schottky junctions within the nanowires, a nickel layer was deposited, patterned and subsequently annealed, promoting nickel intrusion into silicon and the formation of metallic NiSi_2_ segments with atomically abrupt and flat junctions towards the pristine SiNW (Weber et al., 2006; Figure 1C). This process allowed for precise positioning of the metal-NiSi_2_/intrinsic-silicon interfaces within the nanowires at sub-micron resolution. The residual intrinsic silicon channel, together with the two junctions, resulted in a transistor channel length of approx. 1 μm.

Additional details on the fabrication process can be found in the Supplementary Material.

### Electrical Characterization

The chip carrier was plugged into a custom-made FPGA device, controlled by a computer running LabView software. The entire set-up is compact and portable for carrying out experiments in host laboratories. Electrical measurements in liquid were performed in 1× phosphate-buffered saline (PBS) solution with a reference gate voltage, V_*LG*_, directly applied to the liquid via an Ag/AgCl reference electrode, and biased common source- and drain-contacts, which entailed a bias voltage V_*D*_ across the channel (Figure 1F). The V_*LG*_ (V_*D*_) sweeps were recorded per row: each of the 32 rows of transistors was measured at the same time through the 32 independent channels. Starting from the top-most row, the entire array was scanned. Each V_*LG*_ (V_*D*_) sweep took 20 (5) seconds, and it took approx. 10 (2.5) minutes to map the entire sensor matrix. The PBS solution was made from dissolution in water of commercial tablets (VWR, Darmstadt, Germany) with a predefined mixture of salts, resulting in a concentration of 137 mM NaCl, 2.7 mM KCl, and 10 mM phosphate buffer.

### Functionalization

The ALD dielectric (HfO_2_) shell covering the silicon nanowires was functionalized by DNA aptamers that were selective to DA. For the functionalization, a protocol modified from Gang et al. (2015) has been applied, First, the amount of hydroxyl groups on the nanowire surface was increased by an air-plasma activation step. Next, the chip was immediately placed in a desiccator, connected to a vacuum pump, to conduct a gas phase silanization. An open container with 3-(triethoxysilyl)propylsuccinic anhydride (TESPSA) was used as silane source, whose evaporation was facilitated by heating with an external infrared lamp. After 4 h of reaction, the chip was rinsed with isopropanol, dried with N_2_, and incubated for 30 min at 120°C to ensure complete dehydration.

Contact angle measurements on reference planar silicon wafers, coated with HfO_2_, were used to confirm successful surface modification, after application of the same silanization method as for the chip with SiNWs. The sessile drop method with an OCA contact angle meter (Dataphysics, Filderstadt, Germany) was used. The angle increased from 38° for bare HfO_2_ to 56° after TESPSA deposition.

A drop of aptamer in 1× PBS solution was deposited on the chip surface and left to dry during 1 h. The aptamer, synthetized by Eurofins Genomics (Ebersberg, Germany), was a single-strain DNA with sequence 5’-GTC TCT GTG TGC GCC AGA GAC ACT GGG GCA GAT ATG GGC CAG CAC AGA ATG AGG CCC-3’ (Li et al., 2013), and modified at the 5’-end with an amino group. This group reacts with the succinic anhydride from the TESPSA, by a ring opening process forming an amide bond. After rinsing with PBS containing 0.05% Tween 20 to remove loosely bound aptamers, the remaining free surface was blocked by incubation in BSA during 30 min.

The biorecognition between the aptamer and DA was established at nM and μM concentrations using a colorimetric technique (see Supplementary Material).

### Dopamine Sensing

The measurement procedure was as follows. A 1X Phosphate Buffered Saline (PBS) solution containing the target DA concentration ([DA]), at pH 5.5 to prevent DA oxidation (Herlinger et al., 1995; Pham et al., 2009) was incubated on the chip surface for 15 min. After that, the solution was removed by a pipette, and the surface was washed with a clean 1× PBS buffer solution to remove unbound DA. A drop of 0.01× PBS was then casted on the surface and used for electrical characterization of the sensor matrix by V_*LG*_ sweeps at constant V_*D*_. The reason for using a diluted buffer was, once the DA was attached to the aptamer, to increase the Debye length (and detection volume) to a distance of approx. 7 nm (Chu et al., 2017) from the nanowire surface. This distance would be only 0.7 nm in 1× PBS so that the DA molecules would not be detected, given the length of the silane/aptamer complex. The incubation/recording procedure was repeated for several (DA) values in increasing concentration order.

## Results

### Electrical Characteristics of the SiNW SJ-FETs

The final array featured a sensor pitch of 100 μm and an area of approximately 1 μm × 5 μm per sensor element, although the actual active sensing area per sensor was estimated to be approx. 0.4 μm^2^ considering a NW diameter of approx. 20 nm and an average number of 20 NWs per transistor element.

**FIGURE 2:**
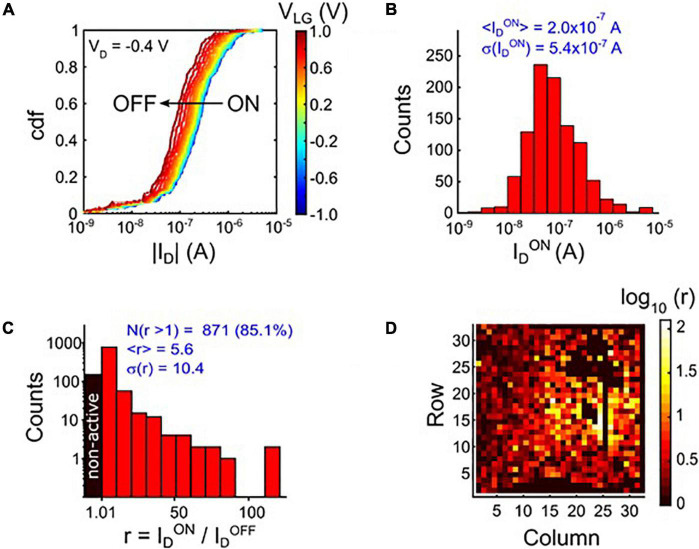
**(A)** Cumulative distribution function (cdf) of the drain currents for the entire ensemble of 1024 devices, measured row by row (32 pixels at a time). Measurements are shown for several values of the liquid-gate voltage, V_*LG*_, extracted from transfer curves. **(B)** Histogram of I_*D*_ in the “ON” state at V_*D*_ = −0.4 V and V_*LG*_ = −0.8 V. **(C)** Histogram of the ratio r between ON (V_*LG*_ = −0.8 V) and OFF (V_*LG*_ = 1 V) current, r = ID_*ON*_/ID_*OFF*_, at V_*D*_ = −0.2 V. The I_*D*_ values are extracted from V_*D*_ sweeps with a constant V_*LG*_. The device yield is calculated as the number of transistors with r = ID_*ON*_/ID_*min*_ > 1. For this chip ∼85% of all devices were found to be active. **(D)** Chip map according to the histogram shown in **(C)**.

We first characterized our SiNW SJ-FETs in liquid phase (Figure 1F) in order to assess their potential as biosensors under physiological conditions. Of special interest were the device-to-device variations. Electrical measurements across the whole array were used to assess the uniformity of V_*LG*_ and V_*D*_ parameters. The measurement data can be used to pre-calibrate and normalize all sensor devices in a buffer solution. Since the readout electronics are integrated on chip, this pre-calibration can be done in an automated manner. Device-to-device variations of the number of wires per transistor result in a spread of I_*D*_, evidenced by the cumulative distribution function (cdf) in Figure 2A and the histogram of I_*D*_ at fixed V_*D*_ (Figure 2B). We defined the number of devices with ID_*ON*_/ID_*OFF*_ >1 as the chip yield of functional devices. Yields of up to 85.1% of all devices were measured with four separate chips (Figure 2C). The map of Figure 2D shows ID_*ON*_/ID_*OFF*_ for all the transistors. Variation of the device properties as well as the presence of non-active devices can be seen.

**FIGURE 3:**
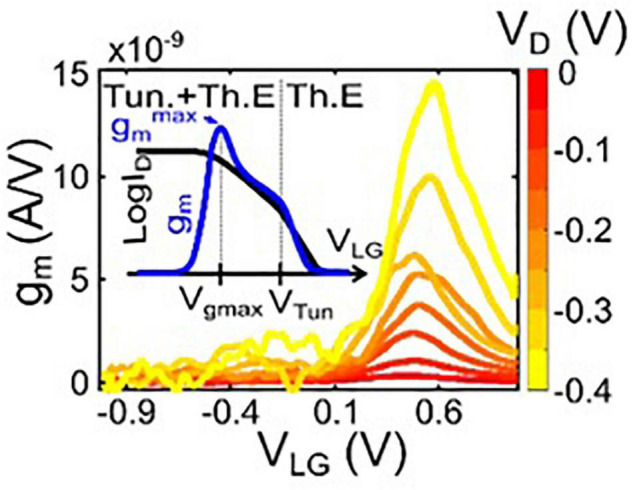
Transconductance g_*m*_ versus drain voltage (V_*D*_) and liquid-gate voltage (V_*LG*_) for one selected device.

Next, we evaluated the SiNW sensor performance and the transport regimes relevant to biosensing. An important parameter for sensing with SJ-FETs is the transconductance, g_*m*_, obtained as the derivative of the transfer curves. Its maximum value is interesting for sensing, because it provides the highest drain current response to a change in the potential of the liquid gate and, thus, indicates the extent of changing surface charge adjacent to the channel. In Figure 3 g_*m*_ is plotted versus V_*LG*_ and V_*D*_. A pronounced peak is observed, whose position and amplitude depend on V_*D*_.

### Dopamine Sensing

**FIGURE 4:**
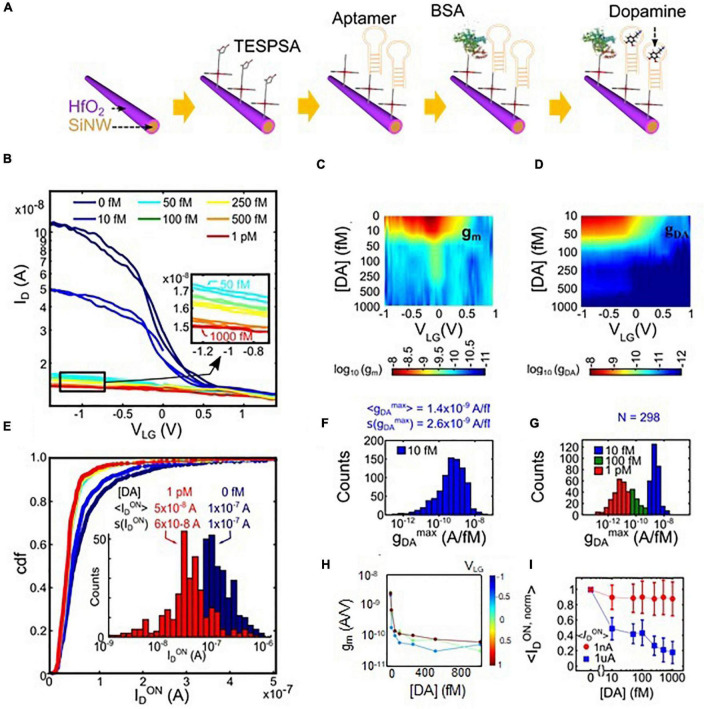
**(A)** Sequence of functionalization steps of the SiNW surface: bare HfO_2_, silanization, aptamer immobilization and blocking with BSA. **(B)** Transfer characteristics of a single device upon exposure to different dopamine concentrations [DA]. The applied drain voltage was always V_*D*_ = 0.2 V. **(C)** Color plot of g_*m*_ (nA/V) as a function of the liquid-gate voltage V_*LG*_ and dopamine concentration [DA]. **(D)** Color plot of g_*DA*_ as a function of V_*LG*_ and [DA]. **(E)** cdf of I_*D*_ in the “ON” state for several DA values. Inset shows the histogram of I_*D*_ comparing the ‘ON’ states at [DA] = 0 fM and [DA] = 1 pM. The measurements were performed at V_*LG*_ = −0.8 V. **(F)** Histogram of the maximum g_*DA*_ value extrapolated for single devices at [DA] = 10 fM and for the corresponding V_*LG*_. **(G)** Histograms of the maximum g_*DA*_ for three [DA] concentration values of a selection of devices for which g_*DA*_ ([DA] = 10 fM) > 10^–9^ A/fM, showing that the highest sensitivity was found in the fM range. **(H)** g_*m*_ versus [DA] at three different V_*LG*_ (high, medium and low) for the same selection of devices as in panel **(C)**, showing that the maximum sensitivity was found in the transistor ‘ON’ region. **(I)** Plot of the average normalized ON drain current ID_*ON,norm*_ measured at V_*LG*_ = −1 V, versus [DA]. The average was determined from two SiNW sensor subsets featuring lower (red circles, ID_*ON*_ approx. 1 nA, subset of 90 SiNW sensors) or higher ID_*ON*_ (blue squares, ID_*ON*_ approx. 1 μA, subset of 7 SiNW sensors). For the second subset, the dynamic range is extended up to 1 pM.

Aptamer-functionalized devices (Figure 4A) were incubated with solutions of increasing DA concentration and their electrical characteristics were measured. In Figure 4B the single-device transfer curves for different DA concentrations are shown in a full forward and backward V_*LG*_ sweep. A dramatic decrease of the transistor source-drain conductance is observed already at concentrations in the femtomolar (fM) range. At high concentrations, an almost complete turn-off of the transistor was observed. DA sensing by SiNW FETs is based on the detection of the charge arising from capturing DA in a functional layer close to the SiNW surface (Lin et al., 2008; Li et al., 2011, 2013).

The current based sensitivity to DA or chemical conductance g_*DA*_, is defined as


$$g_{DA}={\text{I}}_{D}/\delta \left[DA\right],$$

g_*DA*_ decreases with increasing DA concentration (see Figure 4C). The color-map in Figure 4D displays the dependence of the g_*DA*_ on the liquid-gate voltage, and shows that the g_*DA*_ reaches its maximum value when V_*LG*_ correspond to the on-state of the p-type transistor which in the case of the employed Schottky-barrier FETs is dominated by the tunneling regime (Weber and Mikolajick, 2017). This finding suggests that the effect of DA sensing is mostly pronounced, when the carrier injection caused by tunneling and, therefore, relates to the specific working mechanism of the Schottky-barrier FET transducers employed here.

We propose that the presence of DA causes a progressive bending of the intrinsic Si bands, due to the effect of positive charges that are anchored at the nanowire surface and Schottky junction periphery. The high sensitivity and ultra-high measurement resolution to DA results from a direct alteration of the shape and transmissibility of the Schottky barriers, which leads to a blocking of the hole-current injection in the SiNW channel.

Through evaluation of the maximum values of g_*DA*_ and g_*m*_, we can extrapolate from the transfer characteristics that the observed response to a DA concentration change of 1 fM is equivalent to the response to a potential difference ΔV_*LG*_ of 1 V. As discussed in the Summary and Discussion section below, the large response is attributed to the efficient tuning of the charge carrier injection probability through the Schottky junctions, an effect that has a stronger impact then the voltage shift of conventional ISFETs and BioFETs (Weber and Mikolajick, 2017). Due to this strong sensor effect in our experiment, the detection levels are significantly lower than the DA concentrations found in blood or plasma, which are in the pM range (Zeng and Jose, 2011; Robertson et al., 2012). Indeed, the sensitivity is higher than the sensitivity of optical techniques used for bio-recognition (see Supplementary Material), which only can measure reliably in the significantly higher μM range.

Figures 4E–I show the statistical responses of the array to DA dosage, evidencing that the observed behavior for a single transistor is representative for the whole array. In Figure 4E the cumulative distribution function of I_*D*_ is plotted for several DA concentration values, indicating a dramatic decrease of the average I_*D*_ in the presence of DA. The distribution of the maximum of DA sensitivity g_*DA*_ is reported in Figure 4F for the entire array for a DA concentration [DA] = 1 fM. An average value of g_*DA*_*^max^* /g_*m*_*^max^* = 0.6 V/fMwas found for the device ensemble in Figure 4F, and g_*DA*_*^max^* /g_*m*_*^max^* = 0.8 V/fMfor a selected subset of highly sensitive transistors with g_*DA*_ ([DA] = 10 fM) > 10^–9^ A/fM. This result confirms that 1 fM DA produces a similar effect as the substantial V_*LG*_ difference |ΔV_*LG*_| of ∼ 1 V. In Figures 4G,H, the transconductance for a subset of highly sensitive transistors is evaluated. In agreement with single devices, the highest g_*DA*_ was measured for the lowest DA concentration (Figure 4G) and in the transistor’s on-state region V_*LG*_ < V_*gmax*_ (Figure 4H).

### Variability, Specificity, Reusability and Calibration

In the following, the variability, specificity, re-usability and calibration of the SiNW sensing platform will be detailed. A comprehensive DA sensor characterization has been shown for one single chip. The chip, however, features a large number of sensors, and the decrease of I_*D*_*^ON^* with increasing DA concentration has been reproduced on many sensors.

#### Variability

**FIGURE 5:**
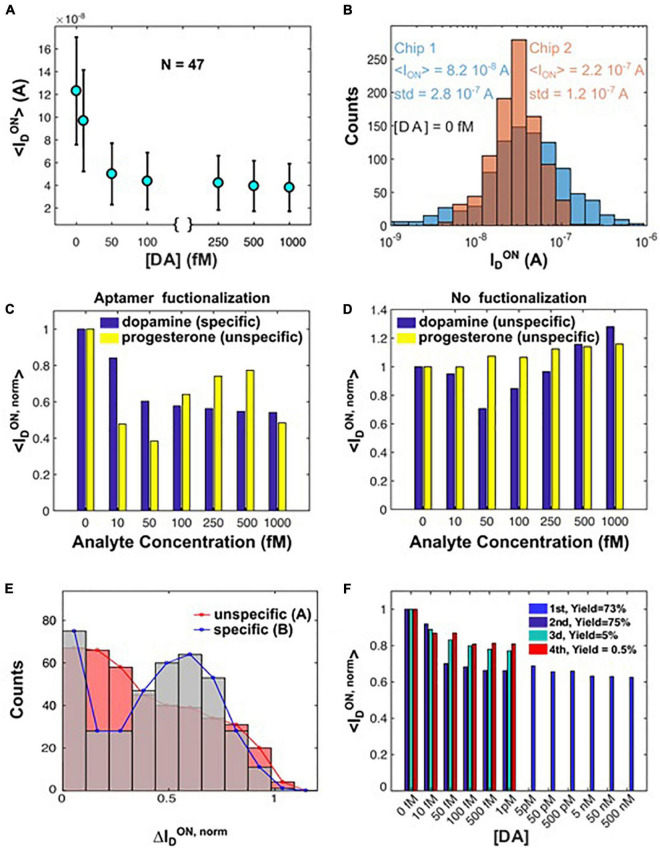
**(A)** Average ON drain current versus [DA] for a small subset (N = 47) of SiNW sensors with similar ID_*ON*_ in the initial state without DA. ID_*ON*_ was extracted from the transfer characteristics at V_*LG*_ = −1 V. **(B)** Chip-to-chip variation of ID_*ON*_ at [DA] = 0 fM. **(C,D)** Controls: for a selection of sensors, response to dopamine and progesterone extracted from transfer characteristics, with **(C)** and without **(D)** aptamer functionalization. **(E)** Sensor response to [DA] = 1 pM, for unspecific (sample area “A”) and specific (sample area “B”) functionalization. **(F)** Reusability: normalized ON drain current for each run of four consecutive dopamine sensing experiments, performed on the same chip. After each cycle the chip surface was cleaned and freshly functionalized with aptamer.

The sensor-to-sensor variability, observed in the previous paragraph, arises from the bottom-up nature of the sensor fabrication and is mostly due to the variable number of SiNWs per device, inherent differences in channel dimensions and to slight differences in the nature of the silicide and in the channel lengths. These variations affect the electrical characteristics of the SiNW transducers, in particular the ON drain current ID_*ON*_ and the transconductance g_*m*_. More homogeneous sensor characteristics are observed when comparing an ensemble of 47 sensors with similar properties, for example similar ID_*ON*_, as in Figure 5A. A comparison of ID_*ON*_ for two different chips after aptamer functionalization can be seen in Figure 5B, showing a similar ID_*ON*_ distribution for both chips. Here and in the following measurements ID_*ON*_ has been extracted at V_*LG*_ = −1 V.

In Figure 4I the average, normalized ON drain current,


$$I_{D}^{ON,norm}=\frac{I_{D}^{ON}\left(\left[DA\right]\right)}{I_{D}^{ON}\left(\left[DA\right]=0fM\right)}$$

is plotted versus the DA concentration for two subsets of SiNW sensors. The first nanowire subset has a “low”initial ID_*ON*_ (1.5 × 10^–8^ ± 2.9 × 10^–9^ A), whereas ID_*ON*_ of the second subset is larger (8.9 × 10^–7^ ± 4.7 × 10^–7^ A). For the first subset, DA detection saturates already around 50 fM, while for the second subset with higher ID_*ON*_, the dynamic range extends up to 1 pM. Due to the proportionality between ID_*ON*_ and the number of SiNWs per device (Pregl et al., 2013), we attribute the observed effect to the larger number of SiNWs in the second subset of sensors. Qualitatively, devices with more SiNWs feature more available binding sites, which become progressively occupied with increasing DA concentration. In devices featuring single or only a few SiNWs the available binding sites are already fully occupied at lower DA concentration, which results in a faster saturation of the ID_*ON,norm*_ versus DA concentration curve. These results indicate that the dynamic range can be optimized by increasing the number of SiNWs per device, which can be achieved by a redesign of the layout of the floating and common electrodes that are employed for nanowire assembly.

The reported detection of DA at femtomolar concentrations with a dynamic range up to 1 pM indicates that DA concentration measurements would be possible even after sample dilution. Sample analysis after dilution is of particular interest in the field of health-status monitoring, where only small volumes of human fluids are available (e.g., tears and eccrine sweat). Dilution would also help to preserve sensitivity, as dilution increases the effective Debye length (Stern et al., 2007), and helps to minimize possible nonspecific adsorption from other molecules that may be present in higher concentrations.

#### Specificity

The specificity of the presented biosensing approach has been investigated by performing several control measurements, the results of which are shown in Figures 5C–E. In Figures 5C,D the sensor responses in terms of ID_*ON,norm*_ of four different scenarios are compared: (i) aptamer functionalization, DA sensing (specific binding, correct target); (ii) no aptamer, DA sensing (unspecific binding); (iii) aptamer functionalization, progesterone sensing (incorrect target); and (iv) no aptamer, progesterone sensing (unspecific binding). An analysis of the measurements shows that in the first case, i.e., specific binding and presence of the correct target (Figure 5C), the sensor response to DA decreases monotonically and its value can be directly correlated to the target analyte concentration, with an average signal decrease of about 50% of its initial value at a DA concentration of 1 pM. This signal change is qualitatively in agreement with previously reported specific DA sensing experiments with inversion-channel SiNWs (Li et al., 2013).

Unspecific DA sensing (Figure 5D) as well as experiments with nonspecific targets (Figure 5C) did not yield any monotonic decay and further did not give direct correlation of the measured signal changes with the concentration of the analyte under investigation.

Finally, the ID_*ON,norm*_ increase up to 20% with respect to the initial signal for increasing progesterone concentrations (Figure 5D) can be explained by nonspecific adsorption of negatively charged molecule species that enhance hole transport in the nanowires.

In a different test, the sensor specificity was investigated by using sensors of the same chip that underwent different functionalization procedures in parallel. The chip area was divided in two sections labeled as “A” and “B.” The transistors in area “A” received the correct surface modification with aptamer (specific functionalization), whereas immobilization of bovine serum albumin (BSA) was performed on the transistors in area “B” (unspecific functionalization). In Figure 5E the statistical distribution of the normalized drain current responses,


$$I_{D}^{ON,norm}=\frac{I_{D}^{ON}-[I_{D}^{ON}\left(DA=0\right)}{I_{D}^{ON}}$$

is compared for the two sections, for a concentration of DA of 1 pM. Transistors in section “A” exhibited a peaked distribution function, similar to a Gaussian, with a peak around approx. 60% of the initial response. The transistors in section “B” yielded a much broader response distribution that peaked around zero response. The non-zero responses were due to non-perfect blocking of the silanes and non-specific interaction of DA with nanowires. These responses could be reduced through optimization of the blocking protocols by adding repelling polymer agents like polyethylene glycol. Such polymers would have the added value of increasing the effective Debye length, improving the obtained signal during the biorecognition process, as described by various previous works (Gao et al., 2015; Haustein et al., 2019).

#### Reusability

The potential re-use of SiNW sensors is demonstrated in Figure 5F. A complete regeneration of the chips was performed, e.g., by removal of the aptamer coating, followed by another functionalization and biosensing measurements. The experiments were performed four times, as evidenced by the bar plot in Figure 5F, which represents ID_*ON,norm*_ versus DA concentration in various runs. The yield of functional devices was approx. 70% for the first two tests and then dropped from the third test on. However, the behavior of ID_*ON,norm*_ was quite reproducible across all runs. Other milder regeneration processes, such as buffer exchange (Lönne et al., 2015) or heat treatment (Wang et al., 2015) to unfold the aptamer to cause a release of the analyte, may lead to even better re-usability. Moreover, the cleaning protocol could be improved. Nevertheless, our data further confirm the reproducibility of the sensing characteristics of the platform.

#### Calibration

**FIGURE 6:**
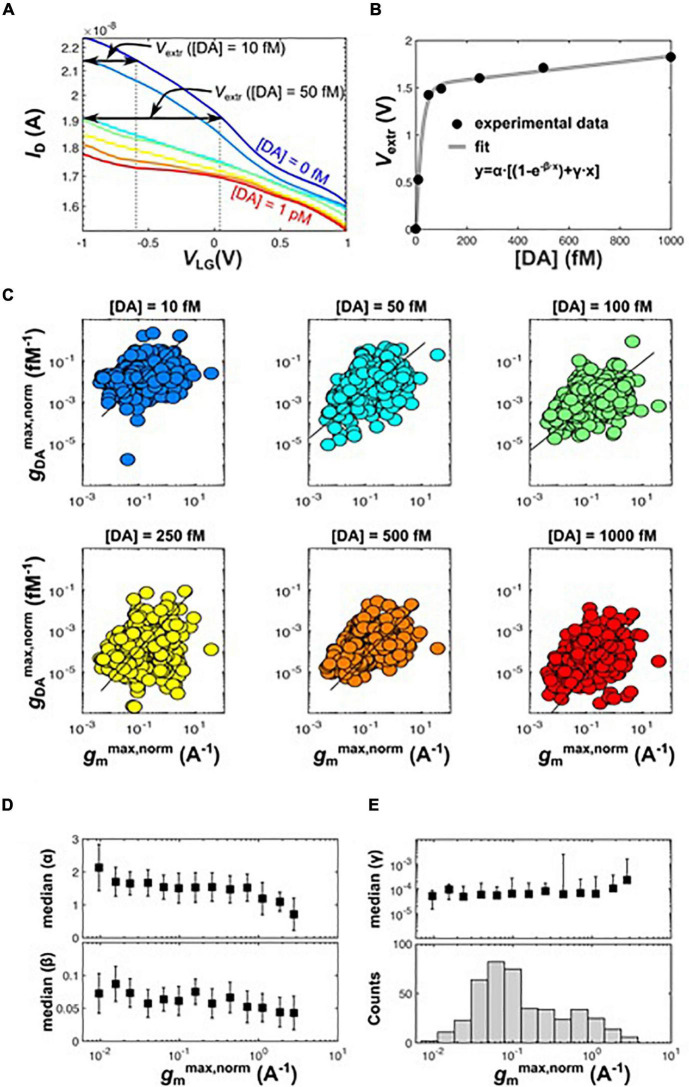
**(A)** Extraction of dopamine sensitivity (V/fM) from single-device transfer characteristics. The ID_*ON*_ ([DA]) at V_*LG*_ = −1 V was used as reference current level. V_*extr*_([DA]) was the voltage difference necessary to produce a drain current variation in the transfer curve without dopamine. **(B)** Plot of V_*extr*_([DA]) versus [DA], representing the specific sensitivity to dopamine of the considered device. Such curves can be used for device calibration. The fit to the data is also shown. **(C)** For several values of dopamine, plots of g_*DA*_*^max,norm^* versus g_*m*_*^max,norm^* was extracted from the “pristine” transfer characteristics, recorded right after aptamer anchoring at [DA] = 0 fM. **(D,E)** Median values of the fit parameters α, β and γ versus g_*m*_*^max,norm^*. Binning was performed according to the histogram in the bottom panel **(E)**.


From single SiNW sensor transconductance curves, recorded at various DA concentration as in Figure 6A, it is possible to extract the biosensor sensitivity to DA in Volts/fM (Figure 6B. The curves also can be used for calibration of the biosensors. The voltage V_*extr*_ ([DA]) corresponds to the liquid-gate voltage change, ΔV_*LG*_, that needed to be applied without DA in order to achieve a drain current change

$$\Delta I_{D}^{ON}=I_{D}^{ON}\left(\left[DA\right]=0\right)-I_{D}^{ON}\left(\left[DA\right]\right)$$

The results of the extraction are shown in Figure 6B for the same transistor as in Figure 6A. The best fit to the data is also shown, corresponding to an exponential plus a linear increase,


$$y=\alpha \left[\left(1-e^{-\beta x}\right)\gamma x\right]$$

where α, β, and γ are the fit parameters.

Taking advantage of the chip reusability, the pre-calibration can be performed upon first usage. This allows to convert the observed ΔI_*D*_*^ON^* changes into corresponding DA concentrations taken at subsequent runs. This recalibration routine can be principally programmed in a much simpler manner than for discrete nano-sensors, since the readout measurement and amplification hardware is already integrated.

Furthermore, prediction of the transistor sensitivity can also be done before DA sensing, by exploiting the transistor parameters introduced in the previous section. In particular, the g_*m*_ recorded in the absence of DA is a very important metric for determining the transistor response to DA and the sensing range, as can be seen in Figure 6C, where a direct correlation between the normalized g_*DA*_*^max,norm^* and g_*m*_*^max,norm^* is displayed. A normalization to I_*D*_*^ON^* ([DA] = 0 fM) has been performed to compare the sensitivity of different devices to cope with device-to-device variability of ID_*ON*_.

In Figure 6D,E the medians of the fit parameters α, β and γ are plotted versus g_*m*_*^max,norm^*. The median is taken in intervals shown at the bottom panel of Figure 6E. Despite the large data scattering, it is possible to recognize a correlation between fit parameters and intrinsic transistor sensitivity to electric charges represented by g_*m*_*^max,norm^*. In particular, α and β decrease for increasing g_*m*_, whereas γ slightly increases. β is the most sensitive fit parameter, since it affects the exponential growth and is inversely correlated to the device sensitivity. α is affected by the overdrive voltage, which is not the same for all devices (i.e., the voltage corresponding to g_*m*_*^max^* varies from device to device).

## Summary and Discussion

A hybrid sensing platform encompassing bottom-up fabricated silicon nanowires on a fully functional CMOS chip containing the readout electronics and signal amplification was developed. Nanoscopic metal (NiSi)-intrinsic-silicon Schottky junctions were fabricated on top of the CMOS chip including source and drain contacts by applying a post-CMOS low-temperature process. A distinct advantage of this approach, is that the high-temperature processing steps needed for the sensor site fabrication (nanowire growth, oxidation and interface treatment anneals) is decoupled from the integration flow encompassing the CMOS chip that only allows for a limited thermal budget. In our SJ-FETs the Si channel is nominally undoped, which simplifies fabrication, as one can circumvent doping-fluctuation issues, and one can rely on distinct analyte-detection mechanisms. Upon selective anchoring of the electrically charged analytes to the bioreceptors, the surface potential at the junction changes, which modulates the width of the Schottky barrier and thus exponentially changes the transmissibility and channel current (Nozaki et al., 2014). In conventional ISFETs featuring ohmic contacts, like most published planar, finFET and nanowire channel geometries, charge coupling to the inversion mode channels only shifts the threshold voltage. In contrast, in our sensor concept, the sensor characteristics and the voltage operation range of SiNW SJ-FETs are altered (Kim and Jeong, 2014).

DA sensing by SiNW FETs is based on the detection of the charge arising from capturing DA in a functional layer close to the SiNW surface. The charged DA molecules produce an additional electric field, E_*DA*_, which acts on the channel and junctions and modifies the transistor’s transfer characteristics. DA sensing reported so far was based on conventional inversion-mode SiNW FET devices (Lin et al., 2008) or planar CMOS ISFETs (Li et al., 2011), where additional charges only led to a threshold-voltage shift. In contrast, the mechanism here is profoundly different and includes a width modulation of the very sensitive Schottky barrier of the SJ-FETs. Thus, the chemical sensing was based on electrical-field tuning of both, silicon-channel properties and transmissibility through the Schottky junctions upon analyte binding. At ultralow concentrations, the binding of charged molecules near the junctions show a stronger impact on the measured current-voltage (I-V) behavior than on the channel itself. This is evidenced in the “quenching” of the transistor on-current as well as in the strong alteration of the entire turn-off behavior characterized by a shallower subthreshold slope in accordance with (Jeon et al., 2015). The change in subthreshold slope causes a strong threshold voltage shift therefore yielding peak sensitivity of 1V/fM for DA with a detection limit in the femtomolar range. Moreover, for high analyte concentrations where the bound charge concentration in the surroundings of the junctions is expected to saturate, the measurementrange dynamically extends over 3 orders of magnitude up to 1 pM. We attribute this to the regular ISFET threshold shift related sensing mechanism in the comparatively large channel region between the junctions.

The possibility to detect analytes in the femtomolar regime can be exploited for minimally invasive health-status monitoring through analyzing external body fluids, such as eccrine sweat, saliva or tears that are only available in small volumes and in which biomarkers typically appear at low concentration levels. Due to the ultra-low detection limit, potential use in waste-water monitoring of specific diseases for control in pandemics may also be considered.

Finally, the correlation analysis of the fit parameters α, β, and γ and the transistor sensitivity evidenced a significant advantage of using a hybrid SiNW/CMOS platform: a forecast of the sensitivity to DA can be attempted based on the gm-values obtained through statistical analysis. As there is a large number of sensor sites, the most suitable devices in terms of sensitivity (fit parameter β small) or dynamic range (fit parameter β large) can be selected in each sector of the chip before introducing the DA solution.

Despite providing a demonstration toward a single analyte, the presence of such high number of sensors could provide a tool for the analysis of a complete biomarker panel. Traditional strategies would not allow the immobilization of the required different receptors for such application given the small sensor pitch. However, a possible solution may be found in electrodeposition processes where reactions can be locally induced by an applied current or potential at the selected sensors (Liu et al., 2014; Ibarlucea et al., 2020).

The developed sensing platform, based on silicon nanowires, provides an interesting route to extend the capability of existing biosensors in terms of detection limit and sensor number and density. The CMOS chip provides the base to exploit the outstanding sensing properties of bottom-up nanostructures on a large scale.

## Data Availability Statement

The original contributions presented in this study are included in the article/Supplementary Material, further inquiries can be directed to the corresponding authors.

## Author Contributions

VS and WW conceived the original idea. VS and BI carried out the biosensing experiments, analyzed the data, prepared the figures, and wrote the manuscript with feedback from all authors. BI and SK performed the experimental biosensing work. BI and LB designed and supervised the biosensing experiments. FS conceived the device fabrication and integration, CMOS measurement setup, electrical characterization, and data analysis. II and NS fabricated the device. AHe characterized it. AHi and UF contributed to the conception of the CMOS setup and integration, as well as to the electrical characterization. WW and GC were in charge of overall direction and project management with input from TM. All authors discussed the results and contributed to the writing of the manuscript.

## Conflict of Interest

NS, TM, and WW were employed by NaMLab gGmbH. UF was employed by MaxWell Biosystems AG. The remaining authors declare that the research was conducted in the absence of any commercial or financial relationships that could be construed as a potential conflict of interest.

## Publisher’s Note

All claims expressed in this article are solely those of the authors and do not necessarily represent those of their affiliated organizations, or those of the publisher, the editors and the reviewers. Any product that may be evaluated in this article, or claim that may be made by its manufacturer, is not guaranteed or endorsed by the publisher.
