# Supplementary material for: Multisite Dopamine Sensing With Femtomolar Resolution Using a CMOS Enabled Aptasensor Chip
Source: Front Neurosci. 2022 Jun 3;16:875656. doi: 10.3389/fnins.2022.875656 (PMC9204155; doi:10.3389/fnins.2022.875656)
Supplement: Supplementary file 1 [file Data_Sheet_1.docx]

Supplementary Material

# Supplementary Data

## *Fabrication*

The CMOS system contains an array of 1024 electrodes individually addressed by 2 x 32 current-mode readout channels. In addition to the foundry process, a passivation using a SiO_2_/Si_3_N_4_ stack was added, and single access points to the underlying electrodes were opened by reactive-ion etching.

In the first step of the integration, we assembled small bundles of pre-synthetized, nominally undoped SiNWs in pre-defined positions by dieletrophoresis (DEP). DEP has been shown to be a powerful and scalable method to assemble and orient, in parallel, large numbers of different nanostructures at desired positions on a custom substrate. (Collet et al., 2015) The applied DEP process simultaneously assembled the NWs in 1024 locations. In our work, a floating electrode was implemented, according to a fabrication route introduced by Seichepine et al. for CNTs (Seichepine et al., 2017), with the aim to achieve large-scale fabrication of independent devices in a single step. Then, 500 nm of Pt were deposited and photolithographically structured to form the common (COM) and dielectrophoresis (DEP) supply rails. Next, the single device leads, the floating (FLO) and the COM extension were fabricated through the deposition of 200 nm Pt and a second lithography and etching step.

Thus, all source electrodes of the individual devices were connected to the COM rails that could be contacted externally to apply an adjustable common-source potential. The counter-electrodes of the devices, FLO, were left floating, but were electrostatically coupled to the nearby DEP rails.

Bottom-up grown, nominally intrinsic SiNWs with 20 nm mean diameter and predominant <112> orientation, synthesized by the vapor-liquid-solid technique (see Methods), were employed. A droplet of a solution of SiNWs, dispersed in isopropanol, was deposited on top of the modified CMOS chip. The DEP NW assembly step was performed by applying an AC signal (20 KHz, 20 VPP) between the DEP and COM electrodes. Due to the small distance of 10 µm, DEP and FLO electrodes are capacitively coupled. As a consequence, SiNWs were captured and aligned in the well-defined 10x5 µm^2^ area between FLO and source electrodes. A specific electrode geometry with flat ends was chosen in order to obtain multiple nanowires, connected in parallel, per device in order to increase the active region and total current while keeping the nanoscopic nature of the nanowires. The process yielded approx. 90% devices where SiNWs connected the contacts.

In the second step of the integration, we fabricated nanoscopic source/drain Schottky contacts on-chip and connected the transistors to the underlying circuits by means of a non-disruptive, low-temperature process. To form the nano-Schottky junctions within the nanowires, a nickel layer was deposited, patterned and subsequently annealed, promoting nickel intrusion into silicon and the formation of metallic NiSi_2_ segments with atomically abrupt and flat junctions towards the pristine SiNW as has been first described in (Weber et al., 2006) (SEM image in **Figure 1(E)** of the main text). This process allows for precise positioning of the metal-NiSi_2_/intrinsic-silicon interfaces within the nanowires at sub-micron resolution. The residual intrinsic silicon channel, together with the two junctions, yielded a transistor channel length of approx. 1µm. The annealing process step was designed to keep the temperature low (350° C for 80 minutes) to prevent metal diffusion and deterioration of the underlying CMOS components and metal layers. After silicidation, the FLO electrodes were connected to the nearest CMOS access points through Pt electrodes, forming the 1024 independent drain contacts. A SEM image of the fabricated SiNW ISFET array at this stage can be seen in **Figure 1(C)** of the main text. The magnification in **Figure 3(D)** of the main text shows a single SiNW ISFET, formed by a parallel arrangement of multiple Si NW channels.

Next, the electrodes and nanowire channels were passivated by a 20 nm thick HfO_2_ dielectric layer by on-chip atomic layer deposition (ALD), which acted as an isolation layer and as the interface layer between the channel and the liquid environment. For enhanced isolation of the outer device leads, a patterned 1 µm-thick SU-8 polymer mask was used. The SiNW/high-k dielectric co-axial structure is shown in Figure 1(F) of the main text. Finally, the chip was wire-bonded to the carrier, and the wires were encapsulated with epoxy for measurements in liquids.

## *Functionalization details and colorimetric test*

In our modified scheme (see **Figure 4(A)** of the main text) an amino-modified aptamer was attached directly to the silane. The use of small receptors like aptamers and short cross-linkers like silanes helps to overcome the Debye length limitation, by capturing the target analyte at a short distance from the nanowire surface.(Chu et al., 2017) The silanization was confirmed by a contact-angle measurement.

The capacity of the aptamer to recognize dopamine (DA) was colorimetrically (Liu et al., 2014) confirmed in microwell plates. First, 50 µL DA in deionized water (DI H_2_O) were incubated with 50 µL aptamer for 30 minutes. During this step, a complex is formed due to the biorecognition process. Then, 50 µL gold nanoparticles (10 nm, British Biocell, Cardiff, United Kingdom) were added and incubated for 30 minutes. The aptamer can adsorb to the nanoparticles when no complex is formed or if no DA is present. Finally, 10 µL of 2 M NaCl were added and incubated for 5 minutes in order to produce salt-induced aggregation of the nanoparticles. When the nanoparticles aggregate, an absorbance peak can be measured at 620 nm. However, if no DA is present or if the aptamer cannot form a complex with DA, the aptamer will adsorb to the nanoparticles, preventing those from aggregation, and the absorbance peak remains at 526 nm. The results are shown in the **Supplementary** **Figure 1**. The wavelength was shifting in the expected direction, obtaining an increased ratio of absorbance values between 620 nm and 526 nm in the nm and µm DA concentration range.


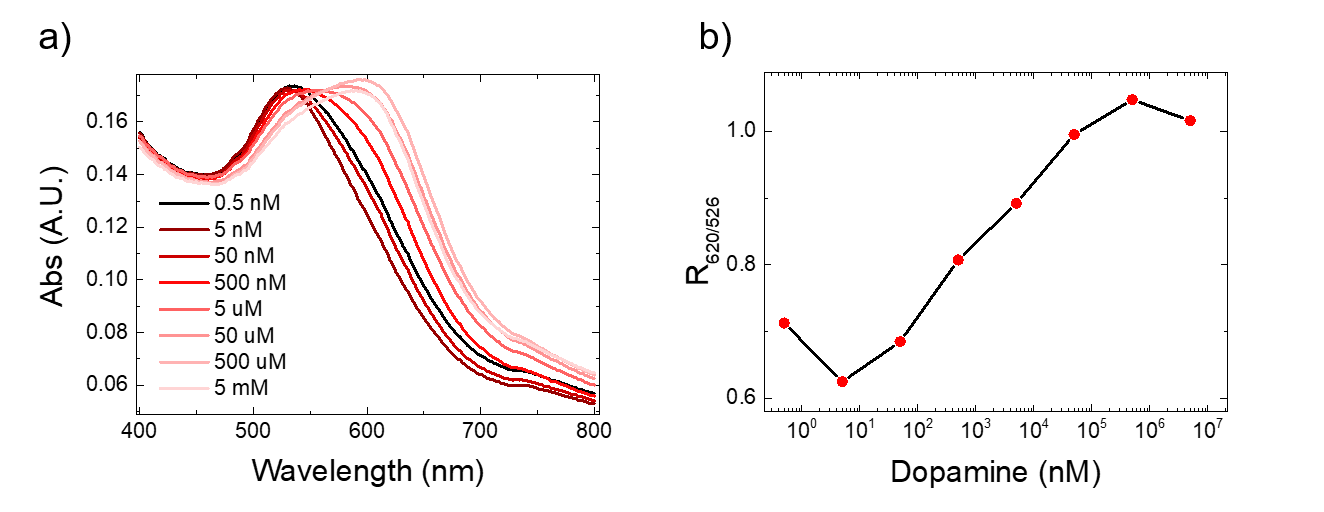


**Supplementary Figure 1**: a) Shift of the gold nanoparticle spectra in dependence of DA concentration; b) Ratio between absorbance values at 620 nm and 526 nm in dependence of the DA concentration.

**References:**

Chu, C. H., Sarangadharan, I., Regmi, A., Chen, Y. W., Hsu, C. P., Chang, W. H., et al. (2017). Beyond the Debye length in high ionic strength solution: direct protein detection with field-effect transistors (FETs) in human serum. *Sci. Reports 2017 71* 7, 1–15. doi:10.1038/s41598-017-05426-6.

Collet, M., Salomon, S., Klein, N. Y., Seichepine, F., Vieu, C., Nicu, L., et al. (2015). Large-scale assembly of single nanowires through capillary-assisted dielectrophoresis. *Adv. Mater.* 27, 1268–1273. doi:10.1002/ADMA.201403039.

Liu, J., Bai, W., Niu, S., Zhu, C., Yang, S., and Chen, A. (2014). Highly sensitive colorimetric detection of 17β-estradiol using split DNA aptamers immobilized on unmodified gold nanoparticles. *Sci. Reports 2014 41* 4, 1–6. doi:10.1038/srep07571.

Seichepine, F., Rothe, J., Dudina, A., Hierlemann, A., Frey, U., Seichepine, F., et al. (2017). Dielectrophoresis-Assisted Integration of 1024 Carbon Nanotube Sensors into a CMOS Microsystem. *Adv. Mater.* 29, 1606852. doi:10.1002/ADMA.201606852.

Weber, W. M., Geelhaar, L., Graham, A. P., Unger, E., Duesberg, G. S., Liebau, M., et al. (2006). Silicon-Nanowire Transistors with Intruded Nickel-Silicide Contacts. *NANO Lett.* 6, 25. doi:10.1021/nl0613858.
